# The MOG antibody associated encephalitis preceded by COVID-19 infection; a case study and systematic review of the literature

**DOI:** 10.3389/fneur.2023.1239657

**Published:** 2023-08-10

**Authors:** Michał Borończyk, Julia Węgrzynek, Agnieszka Borończyk, Joanna Siuda

**Affiliations:** ^1^Students' Scientific Association, Department of Neurology, Faculty of Medical Sciences in Katowice, Medical University of Silesia, Katowice, Poland; ^2^Department of Neurology, Faculty of Medical Sciences in Katowice, Medical University of Silesia, Katowice, Poland

**Keywords:** encephalitis, MOGAD, COVID-19, myelin oligodendrocyte glycoprotein, ADEM-like

## Abstract

**Background:**

New neurological complications of COVID-19 infection have been reported in recent research. Among them, the spectrum of anti-MOG positive diseases, defined as anti-MOG antibody associated disease (MOGAD), is distinguished, which can manifest as optic neuritis, myelitis, or various forms of encephalitis (MOGAE).

**Materials and methods:**

This study reports a new case of MOGAE following SARS-CoV-2 infection. A literature review of other MOGAE cases associated with COVID-19 infection was conducted and summarized.

**Results:**

A 60-year-old male patient, who had previously been infected with COVID-19, was admitted to the Neurology Department with a rapidly progressive deterioration of his cognitive functions that lasted for about 3 months. On neurological examination, the Mini-Mental State Examination (MMSE) score was 17, which further deteriorated to 13. In addition, central paresis of the right VIIth nerve and pyramidal hemiparesis on the right side were noted. The MRI of the brain showed multiple hyperintense lesions. The CSF examination revealed an elevated total protein level with a normal cell count, and serum showed a positive finding of anti-MOG antibodies. Taking into account all the information, the diagnosis of MOGAE, following COVID-19 infection, was made. A total of 9 similar cases of MOGAE associated with SARS-CoV-2 infection were identified in the available literature. Among them 2 cases presented progressive cognitive dysfunction and another 5 altered mental status. The most frequently described MRI changes were hyperintense lesions located cortically and/or subcortically. Anti-MOG antibodies were positive in all patients. In 5 cases they were detected only in serum, in 2 cases in serum and CSF, and in 2 cases the origin was not reported.

**Conclusion:**

The reported cases of MOGAE following COVID-19 infection suggest an increasing new clinical problem, and show an association between COVID-19 and MOGADs.

## 1. Introduction

The COVID-19, which has spread rapidly since the beginning of the pandemic, is caused by severe acute respiratory syndrome coronavirus 2 (SARS-CoV-2). Knowledge about neurological manifestations associated with the infection continues to be updated. The commonly reported symptoms are headache, dizziness, olfactory or tactile disturbances, and impaired consciousness. Rare but more serious complications include acute cerebrovascular conditions, seizures, Guillain-Barré, and Miller-Fisher syndromes, polyneuritis cranialis, oculomotor nerve palsies, meningitis, and encephalitis (1).

Encephalitis is an inflammatory disease that can have various etiologies. Several cases of autoimmune encephalitis (AE) associated with the previous COVID-19 infection have been reported (2–4). Possibly molecular mimicry in response to the virus is thought to lead to the activation of host antibodies that damage the central nervous system (CNS). Several antibodies have been found to attack CNS of patients infected with SARS-CoV-2. The commonly described are anti-NMDAR and others, such as: anti-GAD, anti-CASPR2, anti-amphiphysin, and anti-GD1a/GD1b (5, 6). These also include anti-myelin oligodendrocyte glycoprotein (MOG) antibodies (4, 6), which occur in other demyelinating diseases such as optic neuritis (ON), transverse myelitis or acute disseminated encephalomyelitis (ADEM). The entire spectrum of anti-MOG positive diseases is defined as MOG antibody-associated disease (MOGAD) and affects people of all ages. The incidence is estimated at 1.6–3.4 per million people per year and the prevalence at 20 per million (7).

Although the knowledge about MOGAD is increasing, its association with COVID-19 infection is still insufficient. Therefore, the aim of this study was to present a case of MOG antibody associated encephalitis (MOGAE) following SARS-CoV-2 infection and to review the literature on other MOGADs with similar clinical presentation.

## 2. Case presentation

**Figure 1:**
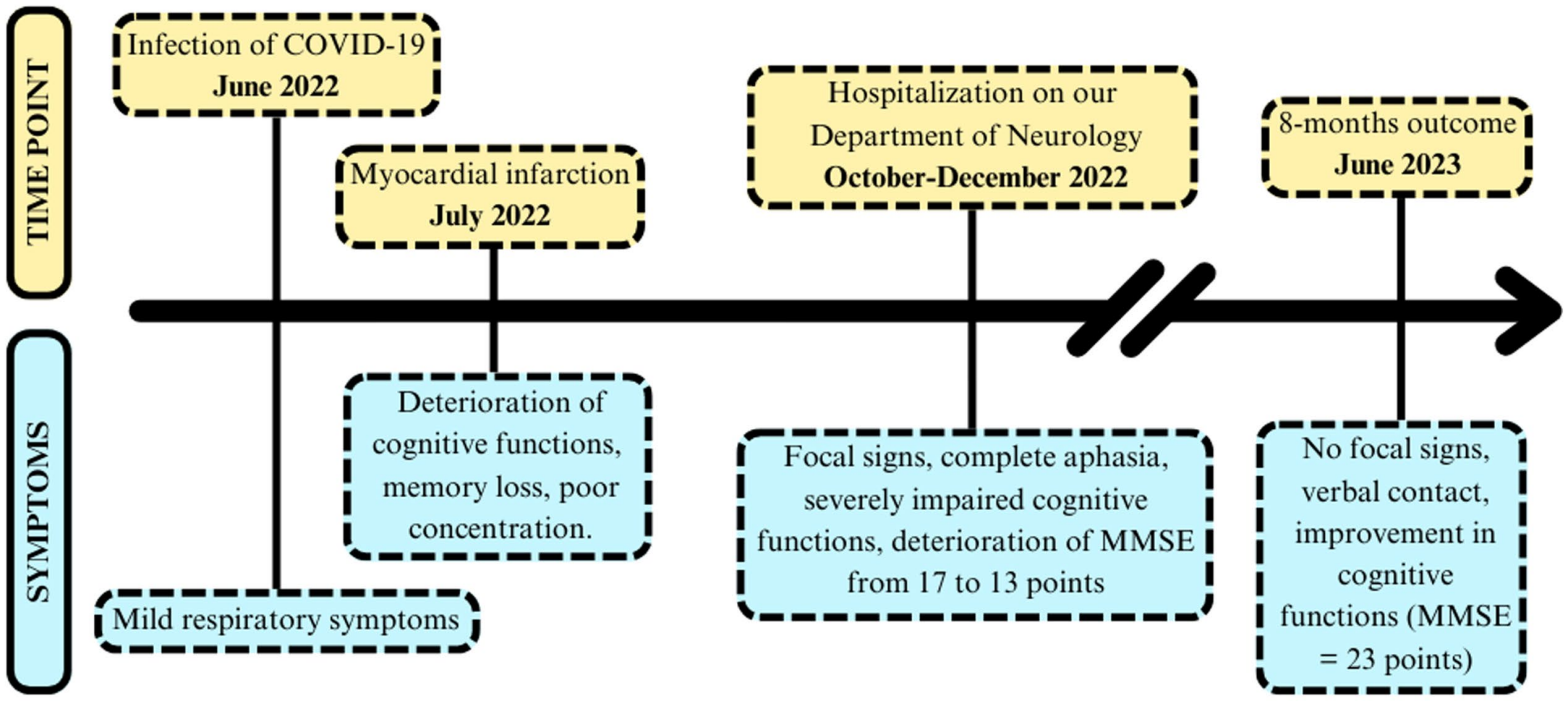
The chronology of symptoms occurrence in the patient.

On October 10, 2022, a 60-year-old patient was admitted to our General Neurology Department with a rapidly progressive deterioration of cognitive functions which lasted for 3 months. Previously, on July 4, 2022, he had suffered a myocardial infarction and was admitted to the Cardiology Department, where he underwent stenting using the percutaneous coronary intervention. On July 20, 2022, SARS-CoV-2 virus was detected by Polymerase Chain Reaction (PCR) method. The only signs of COVID-19 were mild upper respiratory tract infection with fever, so isolation at home was recommended. Three weeks after, initial neurological deficits in the form of memory loss and poor concentration were noted during his stay in the Cardiac Rehabilitation Ward. The chronology of symptoms is presented in Figure 1. Other comorbidities included: arterial hypertension, prostatic hypertrophy, dyslipidemia, chronic heart failure, and permanent blindness of the right eye after toxoplasmosis infection 15 years earlier.

On admission, the patient was in simple verbal contact, self-oriented and disoriented in time and space, Mini*-*Mental State Examination (MMSE) = 17 points. He presented focal signs: the right VIIth nerve central paresis and right side pyramidal hemiparesis. During hospitalization, the patient’s condition worsened in terms of cognitive functions and he also developed complete aphasia. The score of the MMSE decreased to 13 points. On extended psychological examination (the Clock Drawing Test, Five Words Test, Digit Repetition Test, Trace Drawing Test, and Rey Complex Figure Copying Test), significant deficits were found in executive functions, short-term and episodic memory bordering moderate to severe dementia.

**Figure 2:**
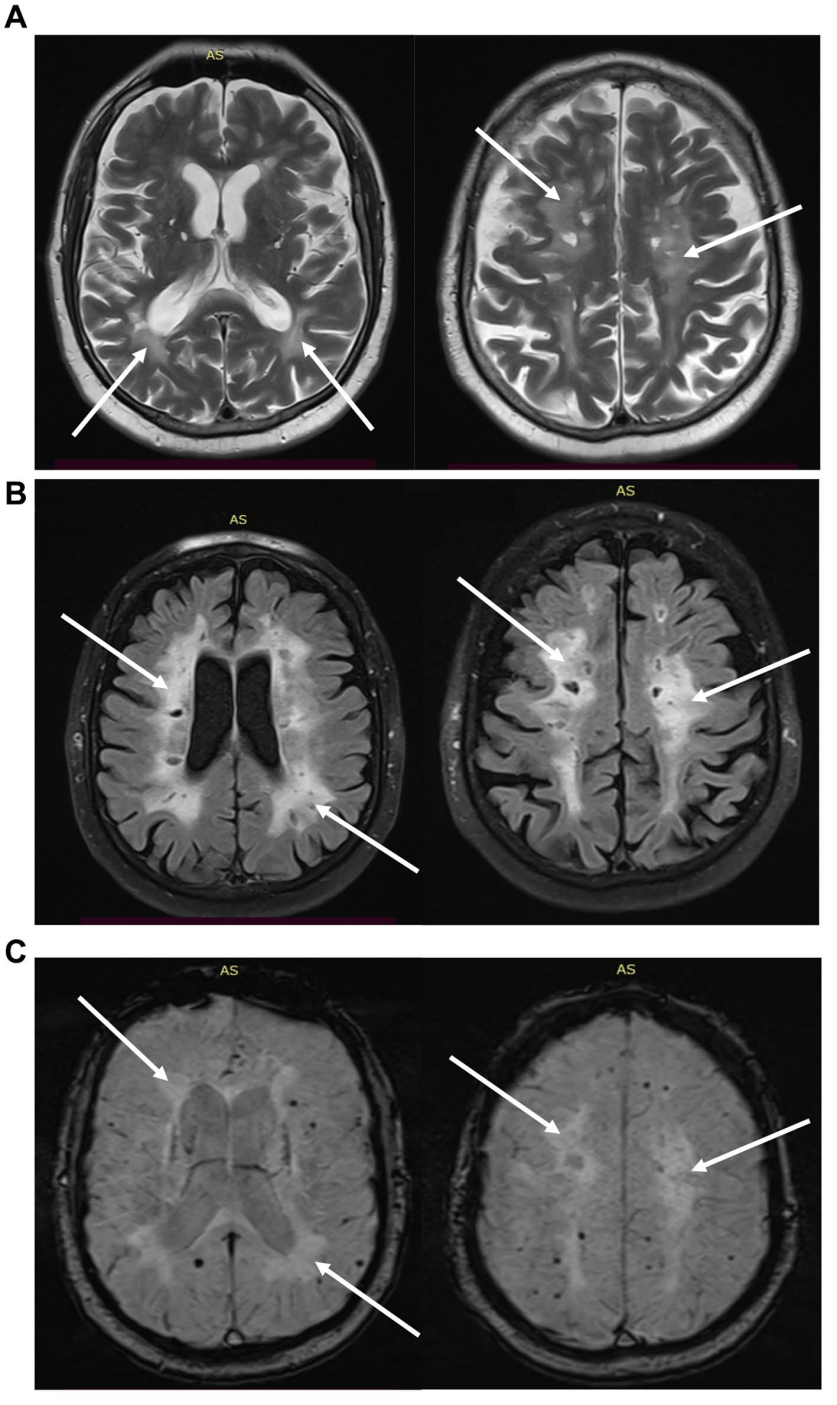
MRI scans of presented MOGAE case showing bilateral T2-hyperintense, poorly demarcated lesions involving supratentorial white matter (solid white arrows). **(A)** T2-dark fluid MRI, **(B)** T2-FLAIR MRI, **(C)** SWI-MRI.

Numerous laboratory tests were performed showing: mild megaloblastic anemia (HGB 12.5 g/dL, MCV 98.2 fl), decreased folic acid level (2.57 ng/mL), slightly increased D-dimmers (821 ng/mL) and lactates (2.72 mmoL/L). Homocysteine and vitamin B12 levels were normal. A head CT revealed numerous hypodense foci up to 11 mm in the periventricular and subcortical white matter. The brain MRI in T2-weighted and FLAIR images showed bands of confluent hyperintense lesions in the white matter of both cerebral hemispheres, periventricularly, in the frontal, parietal, and to a lesser extent in the temporal lobes and corpus callosum, with clear Virchow-Robin spaces on both sides (Figure 2). The described changes were not enhanced by the gadolinium contrast, nor did they exhibit diffusion restriction properties. In addition, numerous vascular changes were noted in the lenticular nuclei. The MRI of cervical spinal cord revealed no abnormalities. The EEG showed generalized slowing of the recording with baseline activity in most leads with an average frequency of 7.5–9.5 Hz, consisting of mixed alpha and theta waves of 6–7 Hz and an amplitude of up to 50 μV. A CSF examination was performed, showing an elevated total protein level (608 mg/L) with a normal cell count (3 cells/μl). The presence of oligoclonal bands (OCBs) was not detected. A comprehensive diagnostic work-up for autoimmune diseases, including a-NMDAR, a-AQ4, auto-antibodies (pANCA, cANCA, a-β2 glycoprotein, ANA, a-dsDNA, lupus anticoagulant 1 and 2), onco- and antineuronal antibodies (a-AMPA1, a-AMPA2, a-GABABR1, a-LGI, a-CASPR2, a-Ri, a-Yo, a-Hu, a-Ma2/Ta, a-Ma1, a-CV2, a-amphizin, a-PCA2, a-Tr, a-SOX1), infectious diseases (*Borrelia*, HIV, HBS, HCV, *M. pneumoniae*, *V. zoster*, VDRL), and genetic diseases (CADASIL, MTHFR gene polymorphism) was performed. A common mutation in the MTHFR gene was found: a heterozygous polymorphism c 677C > T and c 1298A > C. The study of CSF biomarkers revealed an increased total τ- protein level (931 pg/mL), a normal level of hyperphosphorylated τ- protein (41.4 pg/mL), and a normal level of amyloid β-42 (917 pg/mL). In addition, a positive result of anti-MOG antibodies in serum was obtained (titre ≥1:10), by indirect immunofluorescence in duplicate. The presence of anti-MOG antibodies in the CSF was not detected. The results of other autoimmune diseases and paraneoplastic syndromes were negative.

Considering the patient’s overall clinical picture and excluding other possible explanations, the diagnosis of AE was made, according to the Graus criteria (8). Positivity of anti-MOG antibodies determined the MOGAE diagnosis, which was supported by the subsequently published MOGAD diagnostic panel criteria of Banwell et al. (7). Treatment with methylprednisolone at a dose of 1 g i.v. for 5 consecutive days was applied, after which no clinical improvement was observed. It was decided to administer intravenously immunoglobulin infusions (IVIG) in dose of 160 grams (0.4 grams per kg for 5 days). On the 27th day of hospitalization, complete resolution of focal symptoms was observed, the patient was discharged home in a good condition. After 8 months of repeated IVIG treatment (160 grams every 6 weeks), the patient showed inhibition of symptoms progression and a slow improvement of cognitive functions. On the last evaluation, the patient was in full verbal contact with improvement in cognitive testing (MMSE 23 pts). Controlled brain MRI showed neither new findings, nor regression. We believe that the IVIG treatment is effective and it was decided to continue the treatment, so that the full 12 months cycle is completed.

## 3. Literature review

### 3.1. Literature review methods

**Figure 3:**
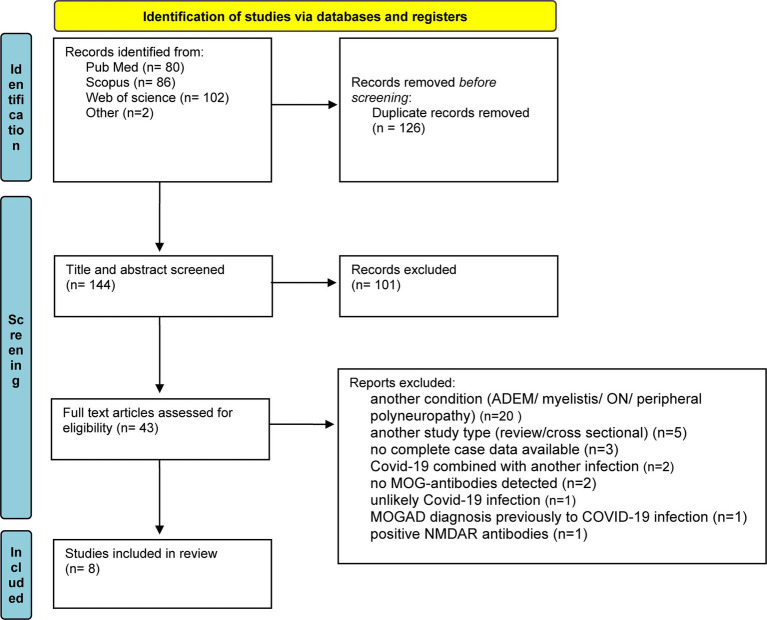
A diagram of screening and identifying similar case studies, following PRISMA guidelines. From Page et al. (9).

We conducted a systematic literature search, according to PRISMA (Preferred Reporting Items for Systematic Review and Meta-Analyses) guidelines, to identify all well-documented cases of MOGAE, preceded by COVID-19 infection. The final search was conducted on June 8, 2023, using search engines: PubMed, Scopus, and Web of Science. The following search terms were used: “COVID-19/SARS-CoV-2” AND “ADEM-like/MOGAD/MOG-associated disease/encephalitis/encephalopathy/case” AND “MOG/myelin oligodendrocyte glycoprotein.” The reference list of these articles was also searched and evaluated for additional reports related to our research, finding 2 more articles. After removing duplicates, we reviewed the titles and abstracts of all results obtained, and finally potentially relevant studies were reviewed in full text. Detailed selection process was shown in Figure 3.

The following inclusion criteria were applied: (1) infection with SARS-CoV-2 virus prior to neurological symptoms, (2) presence of anti-MOG antibodies in serum and/or in the CSF, (3) presence of encephalopathy/encephalitis, with particular emphasis on changes in MRI, without inflammatory changes in other CNS structures (e.g., myelitis, ON, and ADEM), and (4) no other plausible explanation for the above. The following factors were extracted from the selected articles: age, gender, neurological symptoms, days between infection and onset of neurological symptoms, EEG changes, neuroimaging (MRI/CT) changes, abnormalities in CSF, titers of anti-MOG antibodies in serum or/and CSF, treatment applied, and patient outcomes.

### 3.2. Literature review results

The literature search initially found 270 publications, of which 126 were removed due to duplication and 101 were excluded through title and abstract screening. The remaining articles were screened for eligibility using the above criteria. Of the 43 preliminary articles, 20 were excluded, because they described a different condition (ADEM/ON/myelitis/peripheral neuropathy), 5 because they were not a case studies (review/cross-sectional study), 3 because complete case data were not available, 2 because COVID-19 was combined with another infection, 2 because no anti-MOG antibodies were detected, 1 because the MOGAD diagnosis was made before the COVID-19 infection, 1 because COVID-19 infection was unlikely, and 1 because the patient also had positive anti-NMDAR antibodies. A total of 8 articles, including 9 cases, met the inclusion criteria. Five cases were single reports, while four cases were from case series. A summary of the selected articles can be found in Table 1.

**Table 1:** Characteristic of MOGAE cases associated with COVID-19 infection reported in literature.

Case no.	Reference	Gender, age (years)	Neurological symptoms	Days between infection and neurological onset (days)	EEG	Neuroimaging (brain CT/MRI)	CSF abnormalities	Serum/CSF a-MOG tirtle	Treatment	Outcome
1	Durovic et al. (2021) (10)	M, 22	Headache, neck rigidity, general weakness, at 16th day: distinct executive deficits	3	–	**MRI:** multiple disseminated T2 and FLAIR hyperintensities, predominantly cortically	**Pleocytosis:** 31 cells/μl	**Serum:** 1:640 (11th day) and 1:320 (35th day); **CSF:** negative	1 g IVMP per day for 5 days	A full recovery: no residual symptoms, no restrictions, and no medication after 2 months
2	Vraka et al. (11)	F, 1	Altered consciousness, seizures, decorticate posturing, a GCS of 5, episodic right-hand twitching, drowsy, difficulty swallowing	3	Diffuse slow-wave background activity, in keeping with encephalopathy	**CT:** bihemispheric hypodensities, **MRI (4th day):** bilateral widespread white matter hiperintensities in the splenium of the corpus callosum, thalami, and pons	**WBC:** 10/mm^3^	**Serum:** positive **CSF:** negative	IVMP	On discharge, the patient was able to sit, walk a few steps, eat and drink properly, also she had cortical visual impairment (improved after 4 months)
3	Peters et al. (4)	M, 23	**Five weeks prior:** headache, dysesthesias; **Two weeks after testing positive for COVID-19, before admission:** three generalized seizures **On admission:** cognitive slowing, personality changes, worsening, headache, fatigue, mild inattention, delayed recall, decreased verbal fluency	14	Frequent left posterior temporal rhythmic delta activity and epileptiform spikes	**MRI on admission**: normal; **MRI after 2 weeks**: diffuse left-hemispheric cortical T2-fluid attenuated inversion recovery (FLAIR) hyperintensity mostly in the left occipital temporal lobes, left-hemispheric leptomeningeal enhancement	**On admission:** **RBC:** 589/μL, **WBC:** 1/μL; **After 2 weeks: WBC:** 57/ μl (50% lymphocytes, 35% granulocytes, and 15% monocytes); **IgG:** 3.9 mg/dL **IgG index:** 0.84	**Serum:**1:100 **CSF:** negative	1 g IVMP per day for 5 days	Improvement in cognitive symptoms, resolution of headache; 8 weeks after discharge subjective cognitive symptoms resolved completely
4	Ahsan et al. (12)	F, 7	**1 week prior:** two generalized seizures **On admission:** status epilepticus, aphasia, encephalopathy, Todd’s paralysis; **On readmission after 7 days:** headaches, encephalopathy, dysarthria/slurred speech, altered mental status	Same time	Cerebral slowing with left focal slowing	**MRI:** peri Rolandic and posterior parietal lobe restricted diffusion, cortical oedema **MRI at 7th day:** previous diffusion restriction although cortical edema less apparent	**On admission:** **WBC:** 132 cell/mm^3^, **RBC:** 1 cell/mm^3^, **Total protein:** 51 mg/dL **On readmission:** **WBC:** 116 cell/mm^3^ **Total protein:** 48 mg/dL **Multiple OCB** present in CSF/serum	**Serum:** 1:40 (on admission) and 1:100 (on readmission) **CSF:** no data	IVIG 2 g/kg over 3 days	Improvement in condition over 5 days; after observation she had almost returned to her baseline with mild dysarthria
5	Pinto et al. (13)	F, 44	**On admission:** Hand incoordination, weakness, word-finding difficulties, expressive and receptive dysphasia, visual and sensory inattention, MRC-MMT grade 4/5 weakness in the right arm and right leg; **clinical deterioration over next 6 days:** aphasia, no antigravity movements of the right upper limb or at the right hip and knee	7	–	**MRI on admission:** T2-hyperintensity within the centrum semiovale in a periventricular location, temporal and occipital horns, and subcortical deep white matter bilaterally, There was perivascular enhancement. **MRI at 6th day:** progression of the bilateral centrum semiovale and white matter changes, multiple, new cystic spaces without CSF signal, **MRI at 17th day:** residual white matter vasogenic edema but no evidence of residual perivascular contrast-enhanced changes	**WBC:** 13/mm^3^ **Protein:** 50.7 mg/dL, **Glucose**: 2.9 mmol/L (serum glucose 6.3 mmol/L) **CSF at 6th day:** 8 mononuclear cells	positive	1 g IVMP per day for 5 days followed by oral prednisolone 60 mg daily, (PLEX) at 3.5 L/d (1.5 plasma volumes)	A rapid clinical improvement in the neurologic deficit: normal speech, almost full power in the right arm and leg, and no visual or sensory inattention
6	Jacobs et al. (14)	M, 65	**3 weeks prior:** verigo and nonspecific blurry vision **On admission:** altered mental status: patient was disoriented and incoherent, intermittently agitated, combative, and visual hallucinatory	7	**3 weeks before admission:** intermittent epileptic discharges	**MRI 3 weeks prior:** normal **MRI on admission:** multiple FLAIR sequence abnormalities, multiple ring-enhancing lesions on the T1 post-contrast sequence **A follow-up MRI:** the resolution of lesions	**WBC:** 1/mm^3^; **protein:** 52 mg/dL	**Serum:** 1:20 **CSF:** no data	IVMP and IVIG for 5 days	Improvement of symptoms and mentation, correct orientation, no hallucinations
7	Lambe et al. (15)	F, 85	Encephalopathy; seizures	14	–	**MRI:** diffuse, poorly demarcated bilateralcortical/subcortical hyperintensities	**WBC:** 2 cells/mm^3^, **Protein:** 45 mg/dL, **glucose:** 50 mg/dL	Positive: 1:2560	IVMP, ceftriaxone, acyclovir	Death (severe COVID-19)
8	Aubart et al. (16)	M; 10,6	Ataxia, sphincter dysfunction, pyramidal signs	3	–	**MRI:** multifocal and asymmetric cortical lesions	**WBC:** 9/mm^3^ **Protein:** 0.29 g/L **IL1:** 7.5 pg/mL **IL6:** 2.1 pg/mL **TNF:** 41.7 pg/mL	**Serum:** positive **CSF:** positive	none	Complete recovery after 1 month
9	Aubart et al. (16)	F; 4,2	Seizure, impaired consciousness, facial palsy, hemiparesis	No infection symptoms	–	**MRI:** confluent white matter lesions as well as substantia nigra involvement and small cerebellar lesions	**WBC:** 23/ml **Protein:** 0.2 g/L	**Serum:** positive **CSF:** positive	IVMP, pulse of IVIG	Complete recovery after 1 month
10	Our case	M, 60	**3 weeks prior:** memory loss, cognitive impairment **On admission**: MMSE = 17 points; central paresis of the right VIIth nerve and pyramidal hemiparesis on the right side IV grade in the MRC-MMT **During hospitalization:** complete aphasia, MMSE test = 13 points	21	A generalized slowing of the recording consisting of mixed alpha and theta waves	**CT**: numerous periventricular and subcortical hypodensities **MRI:** in T2-weighted and FLAIR images bands of confluent hyperintense lesions in the white matter of both cerebral hemispheres: mainly periventricular, in the frontal, parietal, temporal lobes, and corpus callosum	**Protein:** 608 mg/L	**Serum:** positive **CSF:** negative	IVMP at a dose of 1 g for 5 days, IVIG	**On discharge**: Complete resolution of focal symptoms, cognitively stable **After 8 months:** inhibition of symptom progression and a slow return of cognitive functions. The patient was in verbal contact, oriented in time and space and able to answer simple questions, MMSE = 23 points.

AE, autoimmune encephalitis; CSF, cerebrospinal fluid; CT, computer tomography; EEG, electroencephalography; FLAIR, fluid attenuated inversion recovery; GCS, Glasgow Coma scale; IL-1/−6, interleukin-1/−6; IVIG, intravenous immunoglobulin; IVMP, intravenous methyloprednisolone; MMSE, mini–mental state examination; a-MOG, anti-myelin oligodendrocyte glycoprotein antibody; MRC-MMT, Medical Research Council manual muscle testing scale; MRI, magnetic resonance imaging; no, number; OCBs, oligoclonal bands; PLEX, plasma exchange; RBC, red blood cells; TNF, tumour necrosis factor; WBC, white blood cells.

The documented cases concerned five female and four male patients. Five patients were adults (mean age 47.8 years) and 4 were pediatric patients (mean age 5.7 years). In most cases, except two, infection preceded the onset of neurological symptoms (in one case there were no infection symptoms, and in another case they occurred simultaneously). The most common neurological symptoms were: seizures (5 cases), altered mental status (5 cases), headache (3 cases), general weakness (3 cases), disturbances of consciousness (3 cases), speaking dysfunctions and/or aphasia (3 cases), pyramidal paresis (3 cases), and progressive cognitive dysfunctions (2 cases). An EEG was performed in 4 patients (two adults and two children), showing generalized waves slowing or epileptic discharges.

In one patient, a brain CT was performed, revealing bihemispheric white matter hypodensities. All patients underwent brain MRI, 82% (7 out of 9 cases) demonstrated cortical and subcortical T2 FLAIR hyperintensities, cerebellar involvement was reported in one case, and other case reported diffusion restriction and imaging features supportive of cerebral edema. A CSF analysis was completed for all 9 cases. All had CSF pleocytosis, 6 displayed elevated CSF protein, 1 had positive OCBs and 1 revealed elevated IgG index. In one patient IL-1, IL-6, and TNF were measured, which were slightly above normal.

Anti-MOG antibodies were positive in all patients; in 5 cases only in serum, in 2 cases in serum and in CSF, and in 2 cases the origin of the positive result was not reported. In 5 cases, a specific titre of anti-MOG antibodies was determined, with 3 of them ≥1:100. One patient did not require treatment, others received treatment in the form of methylprednisolone pulses alone or in combination with IVIG (3 cases). Plasma exchange was performed in one patient. Eight patients had a good prognosis after treatment: 4 patients fully recovered, while the remaining 4 improved significantly. One patient died, which might have been caused by complications resulting from severe COVID-19 infection.

## 4. Discussion

MOGAD is an increasingly recognized disease, and its association with COVID-19 has been documented (17). The heightened immune response following COVID-19 infection may provide a potential explanation for this phenomenon (2, 6, 18). In our case, we present a patient who developed MOGAE preceded by COVID-19 infection. Rapidly progressive cognitive dysfunction was the prominent symptom, which has been observed in only a few cases according to literature search (4, 14). However, the mental status and/or consciousness disturbances were observed in five other cases (11–13, 15, 16). These findings are consistent with a recent study by Orozco et al. (19), which demonstrated that cognitive and psychiatric symptoms were prevalent in the majority of patients with AE. Moreover, the presence of focal symptoms has also been reported in patients with encephalitis (20). The brain MRI played a crucial role in establishing the diagnosis. In our patient and in the other cases reviewed, extensive cortical and subcortical changes were observed, which are not typical of other diseases such as Alzheimer’s disease (AD), multiple sclerosis, neuromyelitis optica spectrum disorder (NMOSD), or ADEM (20, 21). Additionally, measuring the concentration of total τ-protein proved helpful, as elevated levels indicate rapid brain tissue damage. Notably, normal concentrations of hyperphosphorylated τ-protein and amyloid β-42 helped exclude AD. Mutations in the MTHFR gene and associated high homocysteine levels have been implicated in the increased cardiovascular risk and various neurological complications, including a higher risk of developing AD (22, 23). However, in our patient, despite the presence of the mutation, homocysteine levels were within the normal range, suggesting that its impact on the clinical picture was negligible.

The presented case was positive for anti-MOG antibodies in serum, but negative in CSF. Following the Graus criteria (8), the antibody assay was repeated, yielding the same result. Additional CSF changes, such as increased protein levels and pleocytosis, were observed in most of the described patients, further supporting the diagnosis (20). The EEG is not included in the diagnostic criteria, since there are no specific changes in the recording. However, a generalized slowing of the recording, as in our case as well as in two other cases (11, 12), or the presence of epileptic discharges were described (4, 14).

Our patient met the criteria for definite AE according to Graus et al. (8). The recently published diagnostic criteria for MOGAD by Banwell et al. (7) provided detailed guidance for the management of patients with positive anti-MOG antibodies. Thereafter, the following criteria were met in this case: the presence of cerebral polyfocal deficits (criterion A), a low positive titre of anti-MOG combined with a negative result for anti-AQP4-IgG and MRI findings expressed as T2 FLAIR supratentorial hyperintensities (criterion B), and the exclusion of other more likely explanations (criterion C).

The treatment administered in our case proved to be effective, as demonstrated by the disappearance of the focal symptoms shortly after the start of treatment and the gradual improvement of cognitive function after several months of IVIG infusions. However, a full evaluation of the patient’s recovery will only be possible after a longer period of time. We are aware that it might be difficult to achieve a complete recovery in our case, because of the patient’s age, coexisting chronic vascular changes, and a long-time distance between the onset of symptoms and the start of treatment. The review found that in 8 out of 9 patients, immunosuppressive treatment with steroids or IVIG proved beneficial and resulted in complete remission of symptoms, further supporting the autoimmune nature of the disease and its hyperinflammatory response after infection by possible molecular mimicry, as previously reported (5).

Herein, we report a case demonstrating a rare occurrence of MOGAE following COVID-19 infection, suggesting a potential overlap between these conditions. The manifestation of rapidly progressive cognitive dysfunction in our patient is an uncommon phenotype in MOGADs, but is consistent with literature findings. The absence of anti-MOG antibodies in the CSF, although consistent with the Graus and Banwell criteria, has been noted in other cases. Distinct MRI findings, not typically observed in other neurological disorders, as well as unique laboratory findings, further support the thesis and suggest a strong association.

### 4.1. MOGAE as an ADEM-like syndrome of MOGAD spectrum

The MOGAD spectrum encompasses a variety of disorders characterized by serum positive anti-MOG antibodies and co-existing inflammation in the CNS (24). The clinical phenotypes description changed in recent years, especially according to MOGAE and ADEM as they represent the common clinical features. ADEM-like syndrome has been described as a group including ADEM, ADEM-ON, multiphase disseminated encephalomyelitis (MDEM), or encephalitis due to the similar clinical presentation (24–26). For the same reason, the clinical criteria for diagnosing any type of AE (regardless of the presence and type of antibodies), published by Graus et al. in 2016 (8), included ADEM. In the review conducted by Lee et al. in 2023, the classification of MOGAE included cortical, limbic encephalitis, and ADEM (27). Knowledge about MOGAD has recently been structured and updated in the criteria proposed by the international MOGAD panel (7). Brain and brainstem involvement in patients with MOG-IgG may manifest as ADEM, cerebral cortical encephalitis, brainstem presentations, or cerebellar presentations. The main difference between ADEM and other diseases is the age of onset; ADEM is most common in children. Cerebral cortical encephalitis, brainstem inflammation, or cerebellitis are rare, and are more likely to occur in adults. The occurrence of seizures appears to be a clinical distinguishing feature between ADEM and non-ADEM conditions (7, 19). From these literature findings, it can be concluded that knowledge about MOGAD needs to be constantly updated due to the fluctuating variations and considerable similarities between some of the disorders mentioned.

### 4.2. Relation between COVID-19 and anti-MOG antibodies

There is an increasing number of reports describing associations between SARS-CoV-2 infection and anti-MOG positive complications (3, 4, 15, 28, 29). Johnsson et al. described four different cases of MOGAD spectrum following confirmation by positive test results for COVID-19. Among these, ADEM, ON, and myelitis coexisting with ON were distinguished (28). Other cases reported by Lambe et al. included multiple cases of ON, acute myelitis, encephalopathies, and brainstem syndrome (15). This strongly suggests that there is an association between COVID-19 infection and emerging MOGADs. Although no statistically significant association was found in the analysis of MOG positive and negative sera, an increase in the monthly distribution of anti-MOG antibody positive cases has been observed since the beginning of the pandemic (30). Several reports suggest that conditions belonging to MOGAD also occur after COVID-19 vaccination (31–33). Among them, cases of post-vaccination encephalitis have been published, such as peduncular and brainstem encephalitis (32), or encephalomyelitis (34). However, it should be noted that anti-MOG antibodies may increase the possibility of an immunogenic process in susceptible individuals; these are very rare cases with a low incidence (31). In light of these literature findings, it is reasonable to suggest that infection may be a triggering factor for MOGAD spectrum disorders. However, further prospective studies are needed to confirm these findings.

## 5. Conclusion

The limited number of reported cases of MOGAE following COVID-19 infection suggests a potential emerging problem. Our case, along with a comprehensive literature review, offers evidence that supports the association between COVID-19 and MOGAE. Such cases, including the one presented, can create diagnostic challenges as they straddle the line between AE and MOGAD. The recent standardization of diagnostic criteria for MOGAD by Banwell et al. (7) may aid in classifying and diagnosing such patients more efficiently.

## Data availability statement

The original contributions presented in the study are included in the article/supplementary material, further inquiries can be directed to the corresponding author.

## Ethics statement

Ethical review and approval was not required for the study on human participants in accordance with the local legislation and institutional requirements. The patients/participants provided their written informed consent to participate in this study. Written informed consent was obtained from the participant/patient(s) for the publication of this case report.

## Author contributions

MB was responsible for the patient case description, discussion, and overall manuscript preparation. JW contributed to the introduction and conducted the literature review on MOGAD. AB conducted the systematic literature review to identify similar cases. JS conceived the study, supervised the project, and provided critical revisions for intellectual content. All authors contributed to the article and approved the submitted version.

## Conflict of interest

The authors declare that the research was conducted in the absence of any commercial or financial relationships that could be construed as a potential conflict of interest.

## Publisher’s note

All claims expressed in this article are solely those of the authors and do not necessarily represent those of their affiliated organizations, or those of the publisher, the editors and the reviewers. Any product that may be evaluated in this article, or claim that may be made by its manufacturer, is not guaranteed or endorsed by the publisher.
